# Future challenges for astrocytology, astroembryology and astrohistology

**DOI:** 10.1038/s41526-026-00600-5

**Published:** 2026-04-24

**Authors:** Kubilay Dogan Kilic

**Affiliations:** 1 Ege University, Faculty of Medicine, Department of Histology and Embryology, İzmir, Türkiye; 2 Helmholtz Center, Institute for Intelligent Biotechnologies (iBio), Munich, Germany

**Keywords:** Biophysics, Cell biology, Physics, Physiology, Systems biology

## Abstract

Human settlement in space brings with it interconnected biomedical challenges stemming from gravity, radiation, and temporal variability. Microgravity alters cellular processes, while cosmic radiation accelerates DNA damage. Furthermore, the absence of Earthly zeitgebers disrupts circadian rhythms and necessitates chronobiological adaptations. To overcome these challenges, interdisciplinary collaboration across physics, medicine, and molecular biology is essential to develop preventive strategies and ensure human health, survival, and evolution beyond Earth’s boundaries.

Sustained human presence beyond Earth, particularly in the context of long-duration missions and possible settlement scenarios, raises a critical biological question: Are we biologically ready? While astrophysicists map the path to other worlds, a critical question remains unanswered: are we biologically ready? Mostofi and Peyravi’s recent correspondence rightly directs our attention to the cellular level^1^. Here, rather than comprehensively reviewing this field, I would like to summarize the potential challenges focused on the cellular level, offer some modest suggestions, and propose terms that should be considered.

Consider our evolutionary paradox: As a species, our journey of toolmaking spans over 3 million years^2^, extending to approximately 3.3 million years when considering the Lomekwi discoveries^3^. Yet, although cytology, histology, and embryology have deep historical roots, including artistic and philosophical traditions extending back more than 3000 years^4^, and modern microscopic biology has advanced rapidly since Leeuwenhoek^5^, our understanding of how cells, tissues, and embryos respond to space-relevant combined stressors remains comparatively immature. Many core principles of terrestrial biology are well established; however, evidence integrating altered gravity profiles, deep-space radiation spectra, and disrupted zeitgebers remains fragmented. In this sense, our engineering skills have advanced faster than our ability to predict biological performance beyond Earth. To survive beyond Earth, we must now learn to master these younger disciplines as skillfully as we wield our tools.

Gravity is the invisible sculptor of human physiology; its absence is a fundamental disruptive factor. However, this challenge is two-sided: it is not only the lack of static weight that reshapes biology, but also the time-varying mechanical loads encountered across mission phases, including transient acceleration/hypergravity during launch and landing, vibration, and other mechanically imposed stresses during transit. Crucially, this challenge is not binary; significant data gaps exist regarding partial gravity environments such as the Moon, Mars, or rotating stations, where biological adaptation remains largely unexplored. While systemic effects, such as muscle atrophy and cardiovascular conditioning loss, are well documented^6^, the risks to cellular dynamics, including cytoskeletal remodeling, mechanotransduction, cell-cycle control and DNA repair run even deeper. Furthermore, microgravity affects cellular processes fundamental to life and reproduction, such as division, repair, and embryonic development^7–9^. These changes underscore the urgency of developing specific subdisciplines such as astrocytology, astrohistology, and astroembryology. Unlike broader space medicine, which focuses on systemic physiology, these specialized fields are necessary to address the microscopic architectural adaptations required for life beyond Earth. Jet pilots exposed to extreme G-forces and increased cosmic radiation provide valuable insights into the physiological adaptations required for space. Studies highlight the need for further research into the combined effects of mechanical and radiation-related stress on the body^10,11^.

Radiation poses an even more insidious threat. Outside the Earth’s protective magnetosphere, astronauts are exposed to high-energy cosmic rays that do more than cause physical trauma; they trigger a molecular cascade. The main enemy here is oxidative stress. Subatomic particles interact with biological matter, generating reactive oxygen species (ROS) and overwhelming the cell’s antioxidant defenses. This oxidative storm not only instantly damages DNA but also accelerates the progression of degenerative diseases and mimics cellular signs of aging^12^. This interaction between radiation and exposure time raises profound questions about the cumulative biological effects of space travel.

Although time is generally defined in physics as a geometric property of space^13^, the concept of temporal organization becomes a critical biological factor when considering long-duration space travel and sustained extraterrestrial habitation. Human biology is deeply connected to Earth’s circadian rhythms, and these rhythms will no longer apply in the changing day-night cycles of extraterrestrial worlds. This disconnect could affect sleep patterns, metabolism, and mental health, requiring a fundamental rethinking of chronobiology. Temporal dynamics also play a vital role in biological processes such as growth, healing, and reproduction. Advances in cryopreservation allow us to slow biological clocks for long-duration space travel, while other technologies may help accelerate biological processes such as tissue repair or embryonic development when needed in harsh environments. Cosmic radiation can increase time-dependent biological deterioration, and the relationship between radiation exposure time and molecular interactions further complicates these dynamics.

Gravity, radiation, and time represent interconnected challenges for humanity’s future in space. To overcome these, we must not only expand our knowledge of astrobiology but also encourage interdisciplinary collaboration across fields such as physics, medicine, and molecular biology. These efforts will not only protect human health but also redefine how we understand life, enabling our advancement within the vast frontiers of space.

## Data Availability

No datasets were generated or analysed during the current study.
